# Identification of the phase composition of solid microparticles in the nasal mucosa of patients with chronic hypertrophic rhinitis using Raman microspectroscopy

**DOI:** 10.1038/s41598-021-98521-8

**Published:** 2021-09-23

**Authors:** Kristina Čabanová, Oldřich Motyka, Hana Bielniková, Lenka Čábalová, Petr Handlos, Dominika Zabiegaj, Karol Zeleník, Jana Dvořáčková, Pavel Komínek, Silvie Heviánková, Miroslav Havlíček, Jana Kukutschová

**Affiliations:** 1 Centre for Advanced Innovation Technologies, VŠB - Technical University of Ostrava, Ostrava, Czech Republic; 2 Faculty of Mining and Geology, VŠB - Technical University of Ostrava, Ostrava, Czech Republic; 3 Nanotechnology Centre, CEET, VŠB - Technical University of Ostrava, Ostrava, Czech Republic; 4 Institute of Pathology, University Hospital Ostrava, Ostrava, Czech Republic; 5 Department of Otorhinolaryngology, University Hospital Ostrava, Ostrava, Czech Republic; 6 Institute of Forensic Medicine, University Hospital Ostrava, Ostrava, Czech Republic; 7 Smart Materials and Surfaces Laboratory, Faculty of Engineering and Environment, Northumbria University, Newcastle upon Tyne, NE1 8ST UK; 8 MEDIN a.s., Nové Město na Moravě, Czech Republic

**Keywords:** Materials chemistry, Environmental sciences, Respiratory tract diseases

## Abstract

Solid particles, predominantly in micron and submicron sizes, have repeatedly been observed as a threat to a human health unique compared to the other textures of the same materials. In this work, the hypothesis the solid metal-based particles play a role in the pathogenesis of chronic hypertrophic rhinitis was investigated in patients who had not responded positively to medication. In the group of 40 randomly selected patients indicated for surgical mucotomy, the presence of solid micro- and submicron particles present in their nasal mucosa was assessed. For comparison, a set of 13 reference samples from patients without diagnosed chronic hypertrophic rhinitis was evaluated. The analysis was performed using Raman microspectroscopy. The advantage of this method is the direct identification of compounds. The main detected compounds in the mucosa samples of patients with chronic hypertrophic rhinitis were TiO_2_, carbon-based compounds, CaCO_3_, Ca(Fe, Mg, Mn)(CO_3_)_2_ MgCO_3_, Fe_2_O_3_, BaSO_4_, FeCO_3_ and compounds of Al and Si, all of which may pose a health risk to a living organism. In the reference samples, only TiO_2_ and amorphous carbon were found. In the control group mucosa, a significantly lower presence of most of the assessed compounds was found despite the longer time they had to accumulate them due to their higher mean age. Identification and characterisation of such chemicals compounds in a living organism could contribute to the overall picture of the health of the individual and lead to a better understanding of the possible causes not only in the chronic hypertrophic rhinitis, but also in other mucosal and idiopathic diseases.

## Introduction

The micron- and submicron particles are continuously observed as a unique toxicological entity. Such materials are often characterised by their particle size—similar to the size of cells or subcellular structures, large specific surface and the surface disruptions resembling the active sites of enzymes, proteins and antibodies. These properties give a new way of toxicity even to the materials which are not recognised as harmful in bulk or in solution. The continuing revelations of newly observed risks for human and public health connected with the presence of micron- and submicron particles in the environment raise legitimate concerns about their management. According to the World Health Organisation (WHO), ambient air pollution contributed to 7.6% of all deaths in 2016 worldwide. The WHO data show that 9 out of 10 people breathe air containing pollutants in high concentrations, leading to 7 million excess deaths worldwide annually. Dispersed in air particles, they pose a serious risk to human health—an increasing number of associations have been reported between the air particulates and a range of diseases^1^, causing adverse health effects such as increased incidences of strokes, Alzheimer’s disease, respiratory and cardiovascular diseases, and cancer^2^.

Moreover, with the developments in industries and transport and the progress in pharmacology and medicine, the risk of environmental exposure of humans to these particles increases^3^. Hence, it is widely believed that humans, living in the close neighbourhood to highways and roads^4^, in urban and industrial areas, are exposed to such particles on a daily basis^5^.

Naturally, the risks related to the harmful nature of the micron- and submicron particles differ significantly with the specific exposure route. The contact with these particles through the digestive system (ingestion exposure) and skin (dermal exposure) requires the mere presence of the particles in either the environment or food and, therefore, are of particular concern. On the other hand, the healthy human body is protected from such exposure by a number of natural factors (compactness and hydrophobicity of skin, stomach acid, gastrointestinal and liver barrier, dermal barrier). Despite that, the adverse effects of the dermal and ingestion contact with micron- and submicron particles have been recorded. The most significant risk to the integrity of the human body has the direct intravenous exposure to the micron- and submicron particles, yet the possible ways of this exposure are limited and, compared to the other possible routes, relatively easily manageable. The contact of the micron- and submicron particles with the respiratory system (inhalation exposure), on the other hand, is practically inevitable in contemporary environments^6^. Moreover, the respiratory system does not possess a similar level of natural protection characteristic for both dermal and digestive routes of exposure. Thus, the inhalation exposure is of the greatest concern towards the influence of the micron- and submicron particles to public health^7,8^.

The fate of the micron- and submicron particles in the respiratory system is mostly affected by their dimensionality. The particles larger than 10 μm are caught on the mucous membrane of the nasal turbinates, then moved using the rhythmical movement of the cellular cilia to the oral cavity and usually subsequently swallowed. Smaller particles, mainly those with one dimension about 1 μm, keep suspended in the air flowing to the secondary and tertiary bronchi and, finally, they diffuse to the pulmonary alveoli wherefrom they are gradually removed by phagocytosis or pinocytosis depending on their size^8,9^.

The particles under 10 μm have been linked explicitly to various respiratory diseases and conditions, which is believed to be caused by their relatively long retention period in the respiratory system. Moreover, multiple other respiratory conditions have been suspected of being caused by exposure to ultrafine particles: chronic rhinosinusitis (with/without polyposis) is one of them. According to European Position Paper on Rhinosinusitis and Nasal Polyps 2020, chronic rhinosinusitis is considered to be a syndrome with a multifactorial aetiology resulting from a dysfunctional interaction between various environmental factors and the host immune system. In healthy individuals, the mucosa serves as a relative barrier modulating interaction with the host immune system, promoting tolerance and symbiosis as well as preventing or limiting inflammation. In patients with chronic rhinosinusitis, the barrier is penetrated with resultant chronic inflammation leading to, in many cases, tissue remodelling (e.g. mucosal turbinate hypertrophy, polyposis) and clinical symptoms^10^. In many patients, the cause of chronic inflammation and mucosal hypertrophy is unclear—the rhinosinusitis is idiopathic. As micron- and submicron particles can cause mucosa inflammatory changes, it is possible that they can be a cofactor causing mucosal hypertrophy.

The treatment of mucosal hypertrophy still remains an open question with many variables, subjected to a continuous discussion. As a first-line treatment of hypertrophic inferior turbinate, intranasal steroids are prescribed. In the scenario when the conservative treatment does not produce sufficient effect, surgical treatment is indicated^11–13^. In the patients with the known cause of chronic rhinosinusitis/inferior turbinate hypertrophy (allergy, drug-induced rhinosinusitis), the treatment is mainly causal—such as removing the allergen from the environment of the patient, desensitisation, or cessation of daily decongestant use. In many cases, though, the exact cause of hypertrophy is unclear. We hypothesise that particularly in patients with chronic rhinosinusitis with/without polyposis with unclear aetiology, micron- and submicron particles might play an important role.

The principal aim of the study was to determine the relationships between the compounds found in the mucosa tissue of patients indicated for surgical treatment and selected environmental factors that would lead to a better understanding of the causes of chronic rhinitis/rhinosinusitis in relation to other idiopathic diseases.

## Material and methods

Forty tissue samples of inferior turbinate mucosa were collected from the patients suffering from chronic hypertrophic rhinitis that were nonresponsive to intranasal steroid therapy and indicated for endoscopic mucotomy. Patients were aged 20–78, both males and females, smokers and non-smokers, manual and office workers (Table S1). For comparison, a set of 13 samples was created from cadaveric donors who were not diagnosed with chronic rhinitis/rhinosinusitis and whose nasal mucosa was healthy (Table S2).

### Sample preparation

The tissue samples, mucosa of the inferior nasal turbinates, were obtained by endoscopic “cold-steel” mucotomy under general anaesthesia. The samples were attached to paraffin tablets using sterile surgical needles; the orientation of the mucosa was marked on the tablet (anterior and posterior sides). Then, the samples were submerged to 10% formalin (Fig. S1). After alcohol-xylene dehydration and automated paraffin embedding, 2–4 μm thin sections were cut and mounted onto glass microscope slides. These sections were deparaffinised in xylene and alcohol and were not stained^14^.

### Analytical methods

Raman spectra allowing chemical characterisation of particles/clusters in the samples were obtained using Smart Raman Microscopy System XploRA (HORIBA Jobin Yvon, France). Raman spectra were acquired in the whole range from 100 to 4000 cm^−1^. 100× objective lens was used, which is more suitable for the samples in thin films or fluids dried on a glass slide. The 532 nm excitation laser source (20–25 mW) with the laser spot diameter of approximately 0.5 µm allowing point analysis of particles/clusters was used. The intensity of the laser was regulated with regard to the measured sample—a lower intensity of the laser beam was set due to the potential of damage to organic samples. XploRA device allows 0.1, 1, 10, 25, 50, and 100% intensity of the initial laser beam. The acquisition time and the number of accumulations were set according to each sample to reduce the signal/noise ratio. Grating with 1200 grooves/mm was set. A screen image recorder camera attached to the microscope enabled the visualisation of white-light images and the selection of the area of interest. In addition, the recording of the spectral images (Raman mapping) was performed in a selected region with 1 µm step. Measured Raman spectra were corrected using the LabSpec software of the XploRA™. The baseline correction (polynomial, order 8th), smoothing (Linear Savitsky-Golay filter, 2nd order, 9 points), and marking the position of the Raman bands in the measured individual Raman spectra were performed by LabSpec software^15^.

### Statistical analyses

The data was analysed using Multiple correspondence analysis (MCA) to assess general trends of explained variation^16^.

Additional editing of data has been made on the presumptions of limits of the Raman spectroscopy. Namely, graphitic and amorphous carbon has been presumed as one indistinctive entity instead of their evaluation as two separated items (C). Carbonates as ankerite and calcium carbonate were also included as one separate entity (CO). Sulphates (BaSO_4_ and CaSO_4_) were also considered as one item (SO), as well as ferric oxides (Fe_3_O_4_ and Fe_2_O_3_) labelled Fe and TiO_2_ anatase and TiO_2_ rutile—Ti. The split in detection between anatase and rutile hits can be explained by the highly amorphous nature of titania deposited on a human mucosa. In fact, the local crystalline structure hit by the laser may resemble either the structure of anatase or rutile and the resulting spectrum (either anatase- or rutile-like) is recorded somewhat randomly.

The variables *diagnosis* (rhinitis × controls), *smoking* (smoker × non-smoker), *age*, *sex*, *occupation* (manual × office) and *occupation* (inside × outside) taken as supplementary—they were only to describe the model but not in its construction. The multivariate assessment was followed by bivariate comparisons of all the pairs of variables—t-tests for the numeric-categorial combination and chi-square tests for the categorial-categorial combination. All the analyses were performed in the R environment, FactoMineR package was used for multivariate analysis^17,18^.

### Research involving human participants and/or animals

The study was conducted from September 2012 to March 2014 following the approval by the Institutional Ethics Committee of University Hospital Ostrava (identifier FNO-ENT-Nanoparticles, 2 RVO-FNOs/2013) and registered at ClincialTrials.gov (identifier NCT02270125). The study was performed complying with the Declaration of Helsinki, good clinical practice, and applicable regulatory requirements. Informed consent was obtained from all participants prior to the initiation of any procedures.

### Ethical standards

Experiments comply with the current laws of the Czech Republic, where they were performed.

## Results and discussion

### Raman microspectroscopy measurements

In the tissue samples (nasal mucosa) of patients with chronic hypertrophic rhinitis, the main detected compounds were TiO_2_ (anatase, rutile), amorphous carbon (AC), CaCO_3_ (calcite), Ca(Fe, Mg, Mn) (CO_3_)_2_ (ankerite), Fe_3_O_4_ (magnetite), MgCO_3_ (magnesite), graphite (Gr), Fe_2_O_3_ (hematite), BaSO_4_ (barite), FeCO_3_ (siderite). The compounds of Al and Si were also detected, albeit less frequently (Table S1). In the reference samples of control group, only TiO_2_ (anatase) and amorphous carbon were detected (Table S2).

**Figure 1:**
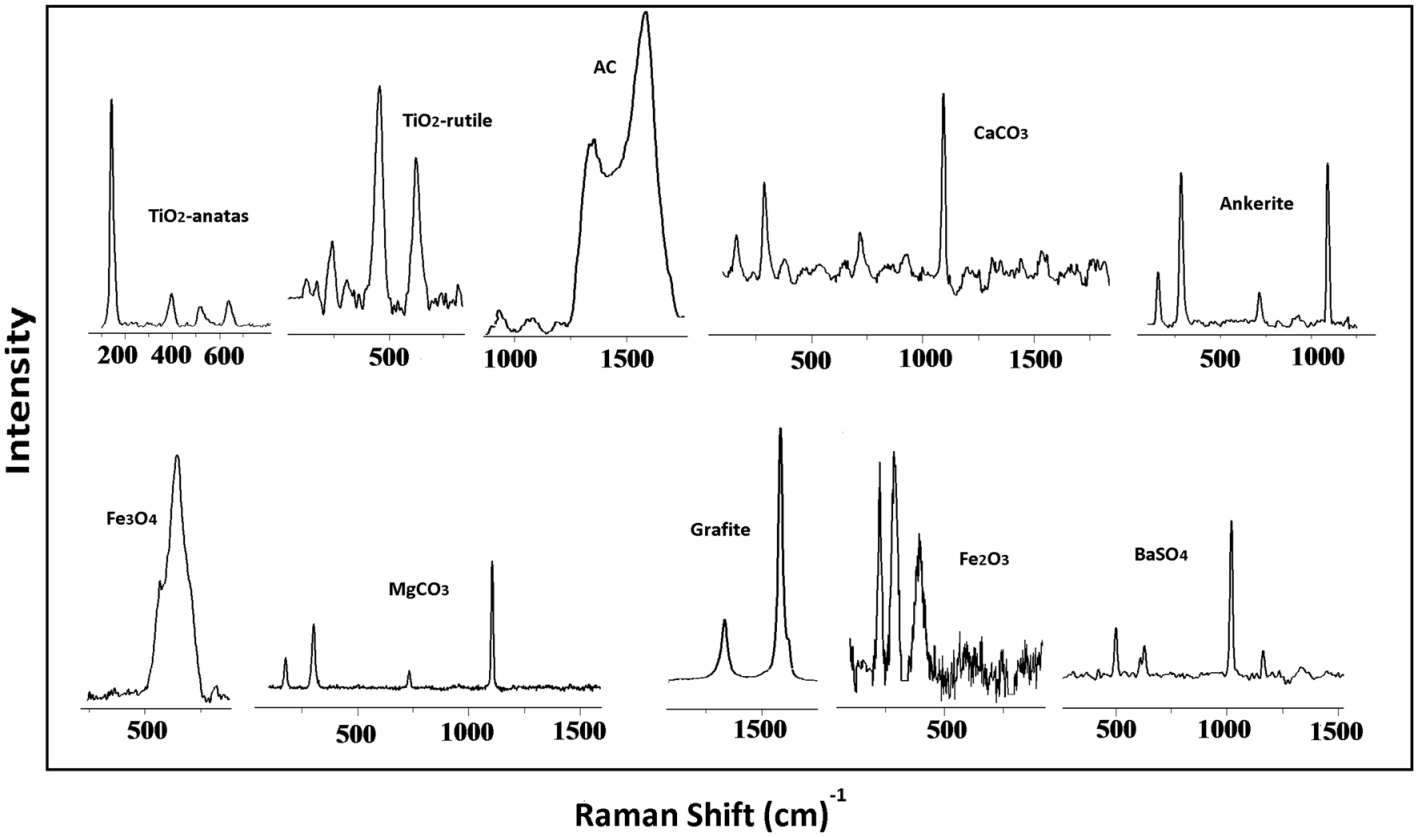
Selected spectra of the compounds found in the hypertrophic tissue samples, measured by Raman microspectroscopy. (TiO_2_-A—anatase, TiO_2_-R—rutile, AC—amorphous carbon, CaCO_3_—calcite, (CaFe(CO_3_)_2_)—ankerite, Fe_3_O_4_—magnetite, MgCO_3_—magnesium carbonate, GR—graphite, Fe_2_O_3_—hematite, BaSO_4_—barite).

The advantage of Raman microspectrometric analysis is the direct identification of compounds (Fig. 1), while it is also possible to get an idea of their size (Fig. S2) thanks to the spectral maps of detected individual compounds. The particles found were mostly in micron sizes, but also as clusters of particles smaller than 1 micron. Submicron-sized particles are highly reactive and tend to agglomerate^19,20^, which could explain the fact that the observed compounds were determined mainly in the form of agglomerates of much smaller particles.

### Statistical analyses

#### The dataset

Patients in the study group (Table S1) have been selected solely on the basis of the indicated mucotomy in University Hospital Ostrava (UHO) and their agreement with providing their tissue samples for the study. Therefore, the common factors of the entire group are (a) diagnosis of the chronic hypertrophic rhinitis unresponsive to the pharmacotherapy and (b) choosing UHO as their hospital of choice (which is usually connected with living in the particular region in the context of the Czech Republic—UHO usually covers the patients from the Moravian-Silesian region). The other features of the group can be viewed as entirely random; therefore, the tendencies observed within the group differing from the properties of the general population in the region might be regarded as the potential risk factors of chronic hypertrophic rhinitis. Also, the chi-square Goodness of fit test confirmed that in the group of patients with chronic hypertrophic rhinitis, the frequency of males differed significantly—was higher—from their frequency in the general population (p = 0.0105). Compared to the frequency of smokers in the Czech Republic (18.2%)^21^, more smokers were found in the assessed group of patients (22.5%); however, the difference was not found to be statistically significant. No statistically significant difference was found by the comparison of the mean age of the assessed group (41.3 years) and the general population in the region (42.2 years)^22^ using a one-sample t-test. Unfortunately, data on the distribution of manual/office jobs neither in the region nor in the Czech Republic are not available; hence, there is no way of knowing whether the occupational pattern differs in any way from the general population. The reference group (Table S2) of patients was selected based on the absence of chronic hypertrophic rhinitis and staying in the Moravian-Silesian region.

#### Multivariate analysis

**Figure 2:**
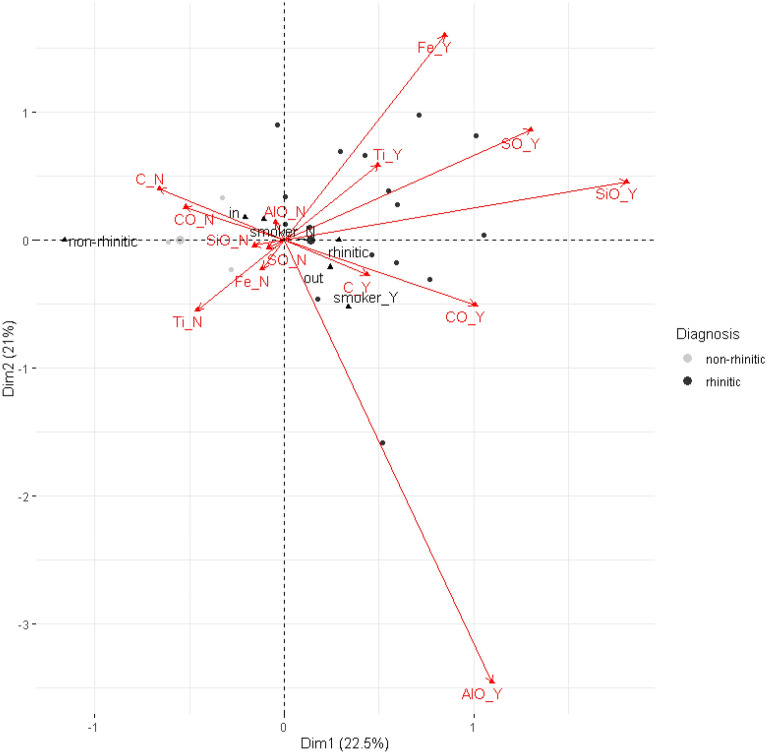
MCA biplot.

Multiple correspondence analysis revealed that the first factor plain retains 43.5% of the total inertia (variation). Biplot of the first factor plain is presented in Fig. 2. From the variables used for the construction of the model, presence statuses of CO (cos^2^ = 0.524), C (cos^2^ = 0.288), and SiO (cos^2^ = 0.284) are well represented by the first axis, AlO (cos^2^ = 0.497) an Ir (cos^2^ = 0.35) by the second axis and Ti by both the first two axes—although to a lesser extent (cos^2^ = 0.227, cos^2^ = 0.318, respectively). The supplementary variables align mostly alongside the first axis, while the most prominent variable is the diagnosis—the only supplementary variable significantly represented by the first axis (cos^2^ = 0.336). Other supplementary variables were not correlated with any of the axes—with the notable exception of smoking status and the second axis.

The results show that there is indeed a difference between the phases found in mucosa tissues from patients with chronic rhinitis and controls. Moreover, this difference is mainly in terms of the confirmed presence of some of the surveyed compounds (CO, C, SiO and Ti) in tissues of chronic hypertrophic rhinitis diagnosed patients. The correlation of the smoking status with the second axis suggests an interesting possible relationship between smoking and presence of AlO, Ir and Ti, although tangential to the diagnosis.

#### Bivariate analysis

A significant association was found for sex and both, occupation and smoking status. In the surveyed group of individuals, the occupation of males was skewed to manual and outdoor labour (p = 0.01); they were also more likely to be smokers (p = 0.02), as revealed by the chi-squared goodness-of-fit test. Smokers in the dataset had CO determined in their mucosa more often (p = 0.04); however, this observation is inseparable from the link between manual/outdoor labour and CO presence (p = 0.01 and < 0.01, respectively)—manual and outdoor workers tended to smoke more (and were, mostly, male). While smokers were clearly overrepresented in the outdoor working patients, the CO presence was overrepresented in smoking individuals among both the outdoor and indoor workers, urban aerosol itself contains a substantial amount of carbonaceous material (20–80%)^23^.

**Figure 3:**
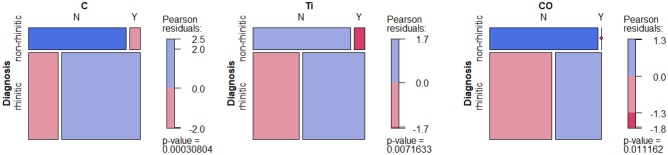
Mosaic plots of C, Ti, and CO association with the diagnosis. Red—underrepresented category, blue—overrepresented category.

With regards to the diagnostic criterion, a significant association was observed for C, Ti and CO as revealed by the chi-squared goodness-of-fit test (p < 0.01, p = 0.01 and p = 0.01, respectively). Rhinitis-diagnosed individuals had the presence of all these compounds determined in their mucosa more often than controls (Fig. 3).

**Figure 4:**
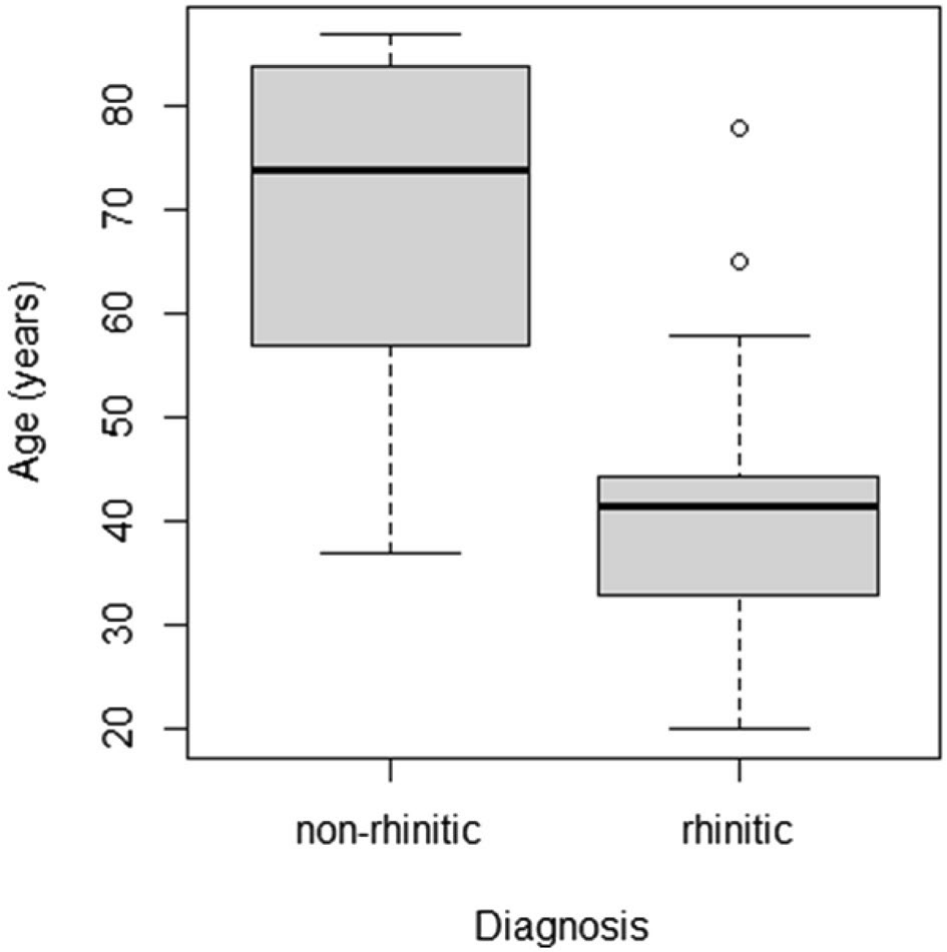
Boxplot of age of rhinitis-diagnosed and non-rhinitis individuals.

Interesting relationship was found between the age of the individuals in the rhinitis-diagnosed and control groups. Controls had a higher mean age (t-test, p < 0.01) than those diagnosed with chronic rhinitis (Fig. 4). In light of the aforementioned association of the presence of certain compounds in the mucosa and chronic rhinitis diagnosis, this means that controls had a lower presence of these compounds in their tissue despite the longer time they had to accumulate them. This was especially relevant for the case of the individuals with C detected in their mucosa who had significantly lower age than those with no presence of C (t-test, p = 0.01). Since there was no association with age and occupational factors, this implies that the tendency to develop chronic rhinitis connected to the accumulation of certain compounds in nasal mucosa may be at least partially innate or due to yet unknown environmental factor. Everyday life exposure should, however, be also considered. On the one hand, the higher age of the control group rules out the presence of particles in nasal mucosa as merely a result of a life-long exposure, on the other hand, it should not be unduly emphasised. Since obtaining the control tissue samples from healthy subjects of comparable age would be unethical, the control tissues came from individuals who died from unknown causes and were, thus, inherently of a higher mean age. At the same time no inflammation or hypertrophy was found in the control inferior turbinate (see Table S2), hence, the comparison strengthens the association between the hypertrophic rhinitis and the observed particles.

The presence of carbon may be related to the ambient air pollution—especially with outdoor/indoor airborne particulate matter containing trace metals, antigens and carbon—mainly in the form of glassy carbon, carbon black or soot. Exposure to particulate matter has been correlated to the living location—namely its vicinity to busy roads and highways leading to elevation of the heavy metal levels in the indoor dust^4^. Amorphous carbon is also present in most printer inks, so the presence of AC (consisting mainly of carbon black) in the mucosa tissue may be related to the release of submicron particles during the printing processes that are commonplace in offices^24^. Road traffic and associated vehicle non-exhausts emissions are also a significant source of many elements released into the environment, e.g. Fe, Cu, S, Zn, Sn, Si, Mg, Al, Ba, and C^25^.

### Health risks

It is not possible to decidedly establish whether were the determined compounds present in the tissues prior to the development of the chronic hypertrophic rhinitis and, thus, led to the chronic inflammation of the mucosa tissue or they entered the tissue when the disease was developed. Microparticles are, by their nature, rather invasive and reactive, hence, they could interfere with extracellular matrix and participate on polyposis formation. However, this process has not been studied so far and is interesting idea for further research in this field.

Nevertheless, many of the detected compounds are likely to be of exogenous origin and with a known negative impact on living organisms. For example, the graphite in the micro- and nano-scale can induce cytotoxicity of LDH (lactate dehydrogenase) and cause a pro-inflammatory reaction of TNF—α (tumour necrosis factor α) on RAW 264.7 macrophages^26^. According to Linberg et al., graphite nanofibers are genotoxic in human bronchial epithelial BEAS 2B cells in vitro^27^. Found iron oxides (Fe_2_O_3_, Fe_3_O_4_) also play an important negative role concerning the human body. Ferrous oxide as fume or dust can cause pneumoconiosis (Siderosis) in the human lung^28^.

There is no known physiological process through which the titania is formed naturally in the human body—and yet the TiO_2_ was present in more than half of the examined samples. All the detected titania is, hence, of exogenic and environmental origin. In the form of nanoparticles, TiO_2_ has several unique characteristics making it a common ingredient in the pharmaceutical and cosmetic industry; hence it is becoming ubiquitous in the human environment^29,30^. Due to the large relative surface area and high oxidation–reduction potential, nano-TiO_2_ is highly reactive, which may negatively impact both environment and human health. Namely, in its micro- to nanostructured form, it is known to interfere with the human body negatively^31–34^. Titanium dioxide nanoparticles may lead to inflammation, fibrosis and lung tumours in humans and are, hence, classified according to International Agency for Research on Cancer (IARC) as potential carcinogens^29,35,36^. They can also have a genotoxic effect leading to (among others) apoptosis or chromosomal instability^37^. Although TiO_2_ is present in many daily necessities (cosmetics, pharmaceuticals, packaging, food) and humans are exposed to it frequently, it was detected only in one case in the reference samples (Table S2).

The limitation of the study is that it cannot be entirely excluded that some microsized particles found in the nasal mucosa in the rhinitis-diagnosed group can come from “contaminated” nasal steroids spray used for the treatment of chronic hypertrophic rhinitis. However, such contamination, especially with the microparticles identified in this study, is not likely. To clarify this issue, it would be interesting to examine inferior turbinate mucosa from patients with chronic hypertrophic rhinitis who were not treated with nasal steroids. However, before surgery, conservative treatment with nasal steroids is always indicated, it would be, thus, unethical to schedule a patient for surgery without the preceding conservative treatment.

Moreover, the conservative treatment with nasal steroid sprays may be inefficient in some patients suffering from hypertrophic rhinitis because the steroid only inhibits the inflammatory response, but it does not affect its cause. If the mucosal turbinate hypertrophy is, indeed, caused by the presence of the particles in the tissue, the microparticle-induced continual irritation may counteract the anti-inflammatory effect of the medication. The presence of the particles in the hypertrophic inferior turbinate may also be an indicator of their presence deeper in the airways or elsewhere in the body of the patient and, hence, underlying microparticle-induced chronic health issues which do not have such easily recognised immediate symptoms as chronic rhinitis but may cause severe health deterioration and decreased well-being in the long term.

Although the association between the development of chronic rhinitis and the presence of particles was observed in the assessed tissue samples, the specific inflammation- and hypertrophy-inducing mechanism is yet to be established. In addition, the links between the presence of microparticles in the inferior turbinate and either their presence elsewhere in the body of the patient or other idiopathic ailments are also worth investigating and should be the aim of further studies.

## Conclusion

The diagnosis status was the key factor explaining the variation in the data when assessed by Multiple correspondence analysis; specific relationships with chronic hypertrophic rhinitis were observed in the cases of amorphous carbon, titanium dioxide and carbonate compounds. All of these compounds were present significantly more in the mucosa of diagnosed patients. Carbonates also seem to be related to smoking and outdoor occupation (which were also interrelated). In contrast to the rhinitis-diagnosed patients, only titanium dioxide and amorphous carbon were found in controls. Interestingly, control group had a significantly higher mean age than the group of rhinitis-diagnosed patients meaning that longer time to accumulate the observed compounds is not a predictor of their presence, while the diagnosis is. Thus, it can be concluded that the presence of these compounds may be linked to upper respiratory system diseases, specifically to chronic hypertrophic rhinitis and, possibly, chronic rhinosinusitis. Whether this link is causal or whether another, underlying cause exists for both of these phenomena has to be investigated in a further study.

Thus, the study shows that many compounds were detected in the mucosa of the patients with chronic rhinitis, namely titanium dioxide, amorphous carbon, calcite, ankerite, magnetite, magnesite, graphite, hematite, barite, and siderite. Since many of these detected micron and submicron particles were shown to have a harmful effect on the human body, the identification and characterisation of such chemicals compounds in a living organism could contribute to the overall picture of the health of the individual and lead to a better understanding of the possible causes not only in the chronic rhinitis with unclear aetiology, also in other idiopathic diseases.

## Supplementary Information

Supplementary Tables.

Supplementary Figures.

## Data Availability

The data presented in this study are available in article and supplementary materials.
