# Supplementary material for: Identification of the phase composition of solid microparticles in the nasal mucosa of patients with chronic hypertrophic rhinitis using Raman microspectroscopy
Source: Sci Rep. 2021 Sep 23;11:18989. doi: 10.1038/s41598-021-98521-8 (PMC8460631; doi:10.1038/s41598-021-98521-8)
Supplement: Supplementary file 2 — Supplementary Figures. [file 41598_2021_98521_MOESM2_ESM.docx]

**Supplementary material for**

# Identification of the phase composition of solid microparticles in the nasal mucosa of patients with chronic rhinosinusitis using Raman microspectroscopy.

ČABANOVÁ Kristina, MOTYKA Oldřich, BIELNIKOVÁ Hana, ČÁBALOVÁ Lenka, HANDLOS Petr, ZABIEGAJ Dominika, ZELENÍK Karol, DVOŘÁČKOVÁ Jana, KOMÍNEK Pavel, HEVIÁNKOVÁ Silvie, HAVLÍČEK Miroslav, KUKUTSCHOVÁ Jana.


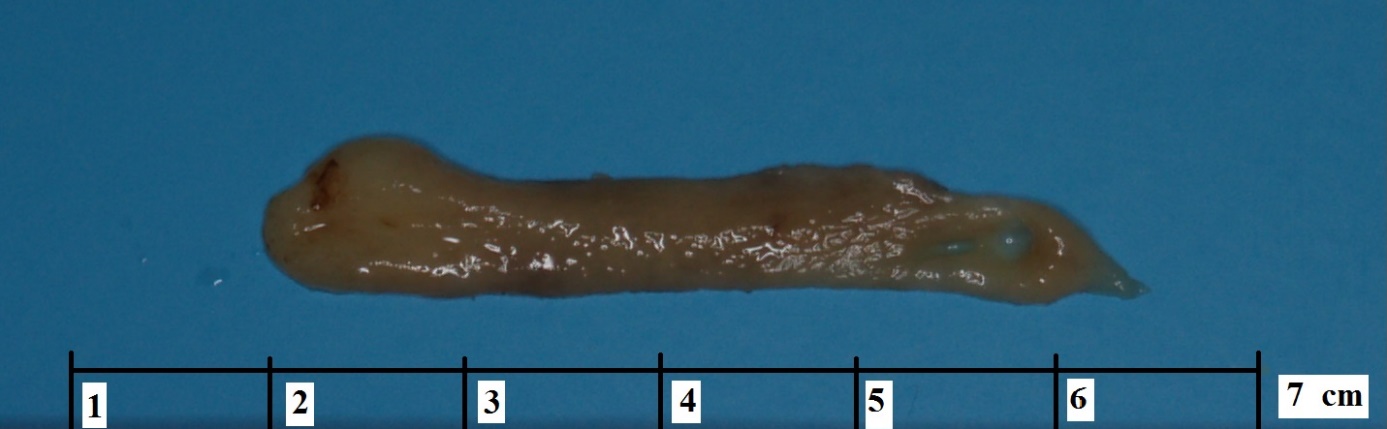


Figure S1: A sample of hypertrophic soft tissue from the inferior nasal turbinates.


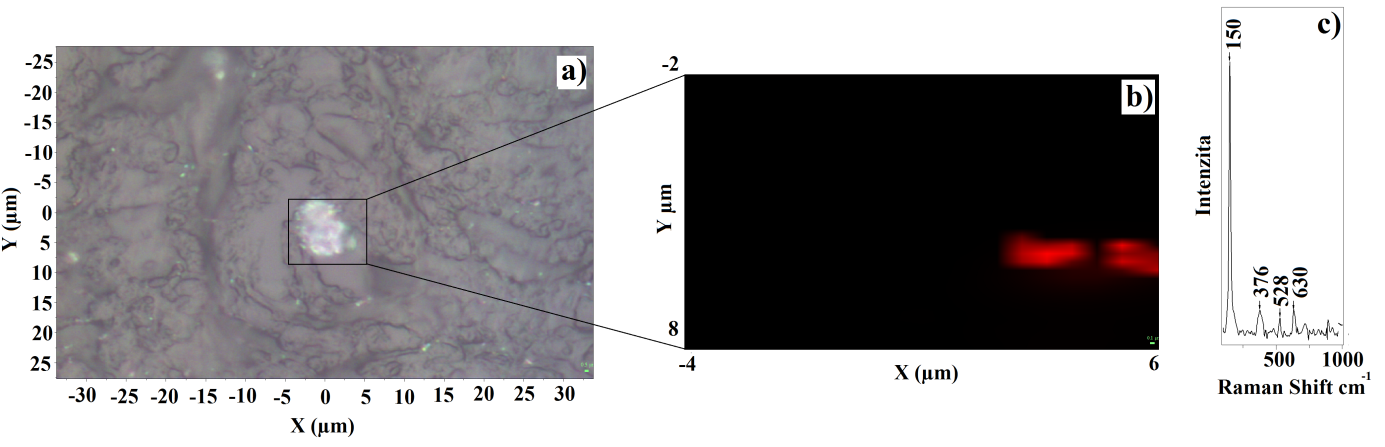


Figure S2: Selected Raman spectral map of the selected area of the hypertrophic tissue (mucotomy, sample M3). a) tissue under a light microscope with the selected area highlighted; b) spectral map of the area (red: sites of TiO_2_ presence, black: surrounding tissue); c) measured TiO2 (anatase) spectrum in the red area.

Table S1: Forty tissue samples of inferior turbinate mucosa from patients with chronic rhinitis: All detected compounds (using Raman microspectroscopy), the anamnestic data of the patients, and histology of all samples.

| sample | sex | age | smoking status | occupation | detected compounds | histology |
| --- | --- | --- | --- | --- | --- | --- |
| M1 | M | 78 | N | welder | AC, ankerite, CaCO_3_, GR, TiO_2_-A | Thickened basement membrane, round cell conglomerates under the basement membrane. |
| M2 | M | 38 | N | programmer | CaCO_3_, Fe_3_O_4_, TiO_2_-A, TiO_2_-R | Cylindrical epithelial hyperplasia, thickened basement membrane, in the stroma: dilation of blood vessels with fibrin clots, occasionally leukostasis. |
| M3 | M | 38 | N | welder | AC, CaSO_4_, Fe_3_O_4_, GR, TiO_2_-A | Thickened basement membrane, cylindrical epithelial hyperplasia, presence of granulocytes, presence of optically active **microparticles.** |
| M4 | M | 65 | Y | policeman | AC, ankerite, GR, TiO_2_-A, TiO_2_-R | Epithelial hyperplasia, thickened basement membrane, glands and stroma with thick-walled vessels. |
| M5 | F | 26 | N | student | Fe_3_O_4_, GR | Numerous inflammatory elements of the lymphoplasmocytic type, thickened basement membrane. |
| M6 | F | 44 | N | labourer | AC, CaCO_3_ | Chronic inflammatory cell conglomerates under thickened basement membrane. |
| M7 | M | 28 | N | student | GR | Presence of optically active **microparticles** in sizes 0.07–0.4 mm in the surface layer of the mucosa, and, dispersively, in the submucosa, chronic inflammatory cell conglomerates under the basement membrane. |
| M8 | M | 44 | Y | labourer | CaCO_3_, GR | Presence of optically active **microparticles** in vessels, chronic inflammatory cell conglomerates under thickened basement membrane. |
| M9 | F | 58 | N | artist | GR, TiO_2_-A | Chronic inflammatory cell conglomerates under thickened basement membrane. |
| M10 | M | 37 | N | manager | GR, TiO_2_-A | Chronic inflammatory cell conglomerates under thickened basement membrane, presence of eosinophils. |
| M11 | M | 42 | Y | builder | GR | Hyperplastic cylindrical epithelium, thickened basement membrane. |
| M12 | F | 45 | N | manager | GR, TiO_2_-A | Hyperplastic cylindrical epithelium with proliferation of goblet cells, chronic inflammatory cell conglomerates with fibrotization under thickened basement membrane. |
| M13 | M | 40 | N | clerk | Fe_2_O_3_, TiO_2_-A | Chronic inflammatory cell conglomerates under thickened basement membrane, presence of granulocytes. |
| M14 | M | 32 | N | policeman | AC, Fe_2_O_3_, GR, TiO_2_-A | Chronic inflammatory cell conglomerates under thickened basement membrane. |
| M15 | F | 53 | N | shop assistant | ankerite, GR, TiO_2_-A | Hyperplastic cylindrical epithelium, thickened basement membrane, in the stroma: hemorrhage and lymphoplasmacytic cell conglomerates. |
| M16 | M | 44 | Y | driver | CaCO_3_, GR, TiO_2_-A, TiO_2_-R | Hyperplastic cylindrical epithelium, stroma with dilated vessels. |
| M17 | F | 42 | N | warehouse keeper | ankerite, BaSO_4_, TiO_2_-A | Presence of optically active **microparticles** above the basal layer of the epithelium, hyperplastic cylindrical epithelium, stroma with dilated vessels. |
| M18 | M | 34 | Y | carrier | AC, CaCO_3_, GR, SiO_2_, TiO_2_-A | Thickened basement membrane, only partially preserved hyperplastic cylindrical epithelium with goblet cells. |
| M19 | M | 48 | N | waiter | ankerite, CaCO_3_, Fe_2_O_3_, SiO_2_, TiO_2_-A | Mucosa with pronounced dilatation of blood vessels, mild hyperemia, chronic inflammatory cell conglomerates under thickened basement membrane. |
| M20 | M | 35 | Y | tinsmith | ankerite, CaCO_3_, GR, SiO_2_ | Hyperplastic cylindrical epithelium with multiplication of goblet cells. |
| M21 | M | 31 | N | labourer | ankerite, GR | Hyperplastic cylindrical epithelium with multiplication of goblet cells, thickened basement membrane, lymphoplasmacytic cell conglomerates, presence of granulocytes. |
| M22 | M | 28 | N | [rolling mill](https://slovnik.seznam.cz/en-cz/?q=rolling-mill) [operator](https://slovnik.seznam.cz/en-cz/?q=operator) | TiO_2_-A | Hyperplastic cylindrical epithelium with the multiplication of goblet cells, thickened basement membrane. |
| M23 | M | 28 | N | operator | TiO_2_-A | Optically active **microparticles** dispersed on the surface or occasionally in the submucosa, chronic inflammatory cell conglomerates under thickened (mildly) basement membrane. |
| M24 | M | 54 | N | policeman | AC, GR | Hyperplastic cylindrical epithelium with multiplication of goblet cells, chronic inflammatory cell conglomerates under thickened basement membrane. |
| M25 | M | 44 | N | executive director | AC, Si comp., TiO_2_-A, TiO_2_-R | Mild chronic inflammatory cell conglomerates with no signs of activity, thickened basement membrane. |
| M26 | M | 42 | N | train dispatcher | AC, CaCO_3_, GR | Optically active **microparticles** in the mucosal epithelium and stroma, in the stroma: chronic inflammatory cell conglomerates, presence of eosinophils. |
| M27 | M | 25 | N | student | AC | Hyperplastic cylindrical epithelium, chronic inflammatory cell conglomerates. |
| M28 | M | 42 | Y | police officer | AC, ankerite, BaSO_4_, GR | Optically active **microparticles** in the mucosal surface and, submucosally, lymphoplasmacytic cell conglomerates under thickened basement membrane. |
| M29 | M | 25 | Y | unemployed | AC, GR, TiO_2_-A | Chronic inflammatory cell conglomerates, thickened basement membrane. |
| M30 | M | 39 | Y | welder | AC, Al comp., (CaMg)CO_3_)_2_) | Presence of optically active **microparticles** in the stroma, chronic inflammatory cell conglomerates. |
| M31 | F | 20 | N | student | GR, TiO_2_-A, TiO_2_-R | Presence of optically active **microparticles**, presence of lymphocytes, plasma cells and eosinophils, mild chronic inflammatory cell conglomerates. |
| M32 | M | 31 | N | administrator | AC, Al comp., ankerite | Clusters of optically active **microparticles** on the mucosa surface, chronic inflammatory cell conglomerates, thickened basement membrane. |
| M33 | M | 43 | N | mechanic | AC, GR, TiO_2_-A | Hyperplastic superficial epithelium with thickened basement membrane. |
| M34 | F | 41 | N | seamstress | GR, TiO_2_-A | Chronic inflammatory cellulization both in the stroma and submucosally, presence of granulocytes. |
| M35 | F | 65 | N | manager | CaCO_3_, TiO_2_-R | Hyperplastic superficial epithelium with a thickened basement membrane. |
| M36 | M | 36 | N | IT technician | GR, TiO_2_-A | Thickened basement membrane, mild chronic inflammatory cell conglomerates. |
| M37 | M | 44 | N | labourer | - | Thickened basement membrane, disperse chronic inflammatory cell conglomerates. |
| M38 | M | 34 | N | electro-technician | AC | Optically active **microparticles** in mucosa, chronic inflammatory cell conglomerates, in the stroma: hyperemia, hyperplastic mucosa with a large number of goblet cells on the surface, thickened basement  membrane. |
| M39 | M | 55 | N | businessman | - | Thickened basement membrane, chronic inflammatory cell conglomerates. |
| M40 | M | 54 | N | bailiff | - | Thickened basement membrane. |

### Al comp. = aluminium compounds; GR = graphite; AC = amorphous carbon; Si comp. = silicon compounds

Table S2: Thirteen samples from patients who were not diagnosed with chronic rhinitis and whose mucosa was asymptomatic. All detected compounds (using Raman microspectroscopy), the anamnestic data of the patients, and histology of all samples.

| sample | sex | age | smoking status | occupation | detected compounds | histology |
| --- | --- | --- | --- | --- | --- | --- |
| R1 | M | 71 | N | ambulance driver | - | Nasal concha histologic sample, cylindric epithelium is nearly completely separated from intact basal membrane. Epithelium cells with no signs of hyperplasia. Stroma with no inflammatory cells and with presence of well formed glands. |
| R5 | M | 57 | Y | miner | - | Fragmented nasal concha histologic sample; stroma with no inflammatory cells and with presence of well formed glands. Cylindric epithelium is completely separated from the basal membrane. Preserved epithelium cells with no signs of hyperplasia. |
| R6 | M | 77 | N | coke oven operator | TiO_2_-A | Congested nasal concha histologic sample, cylindric epithelium with signs of focal hyperplasia with intracellular vacuoles. Basal membrane is thickened. Stroma with focal presence of lymphoplasmacytic cells and well formed glands. |
| R7 | F | 48 | N | office worker | AC | Nasal concha histologic sample, cylindric epithelium is nearly completely separated from intact basal meurembrane. Epithelium cells with no signs of hyperplasia. Stroma with focal presence of lymphoplasmacytic cells and well formed glands |
| R8 | M | 44 | N | locksmith in the mine | - | Nasal concha histologic sample, cylindric epithelium is nearly completely separated from intact basal membrane. Epithelium cells with no signs of hyperplasia. Stroma with focal presence of lymphoplasmacytic cells and well formed glands |
| R9 | M | 67 | Y | labourer | - | Congested nasal concha histologic sample, cylindric epithelium with intracellular vacuoles and with no signs of hyperplasia. Basal membrane is intact. Stroma with well formed glands and with no presence of inflammatory cells. |
| R10 | M | 37 | Y | labourer | - | Congested nasal concha histologic sample, cylindric epithelium with signs of focal hyperplasia with intracellular vacuoles. Basal membrane is thickened. Stroma with focal presence of lymphoplasmacytic cells and well formed glands. |
| R11 | F | 87 | N | office worker | - | Congested nasal concha histologic sample, cylindric epithelium with signs of focal hyperplasia with intracellular vacuoles. Basal membrane is thickened. Stroma with focal presence of lymphoplasmacytic cells and well formed glands. |
| R12 | F | 84 | N | office worker | - | Congested nasal concha histologic sample, cylindric epithelium with signs of focal hyperplasia with intracellular vacuoles. Basal membrane is thickened. Stroma with focal presence of lymphoplasmacytic cells and well formed glands. |
| R13 | F | 84 | N | office worker | - | Congested nasal concha histologic sample, cylindric epithelium with signs of focal hyperplasia with intracellular vacuoles. Basal membrane is thickened. Stroma with focal presence of lymphoplasmacytic cells and well formed glands. |
